# Multi-synchronization and other patterns of multi-rhythmicity in oscillatory biological systems

**DOI:** 10.1098/rsfs.2021.0089

**Published:** 2022-04-15

**Authors:** Albert Goldbeter, Jie Yan

**Affiliations:** ^1^ Unité de Chronobiologie théorique, Faculté des Sciences, Université Libre de Bruxelles (ULB), 1050 Brussels, Belgium; ^2^ Center for Systems Biology, School of Mathematical Sciences, Soochow University, Suzhou, People's Republic of China

**Keywords:** oscillations, biological rhythms, synchronization, birhythmicity, bistability, computational systems biology

## Abstract

While experimental and theoretical studies have established the prevalence of rhythmic behaviour at all levels of biological organization, less common is the coexistence between multiple oscillatory regimes (multi-rhythmicity), which has been predicted by a variety of models for biological oscillators. The phenomenon of multi-rhythmicity involves, most commonly, the coexistence between two (birhythmicity) or three (trirhythmicity) distinct regimes of self-sustained oscillations. Birhythmicity has been observed experimentally in a few chemical reactions and in biological examples pertaining to cardiac cell physiology, neurobiology, human voice patterns and ecology. The present study consists of two parts. We first review the mechanisms underlying multi-rhythmicity in models for biochemical and cellular oscillations in which the phenomenon was investigated over the years. In the second part, we focus on the coupling of the cell cycle and the circadian clock and show how an additional source of multi-rhythmicity arises from the bidirectional coupling of these two cellular oscillators. Upon bidirectional coupling, the two oscillatory networks generally synchronize in a unique manner characterized by a single, common period. In some conditions, however, the two oscillators may synchronize in two or three different ways characterized by distinct waveforms and periods. We refer to this type of multi-rhythmicity as ‘multi-synchronization’.

## 1. Introduction

Together with oscillatory behaviour, bistability represents one of the most conspicuous nonlinear phenomena in biological systems [1–4]. Bistability refers to the coexistence between two simultaneously stable states. In principle, these may be stationary or oscillatory states, but the term is generally used, and will be used hereafter, for the coexistence between two stable steady states, if only because this situation is more common than the coexistence between two oscillatory states, a phenomenon referred to as birhythmicity [5]. The coexistence can involve three stable steady states (tristability) or three periodic regimes (trirhythmicity). The goal of this paper is to focus on the coexistence between two or more oscillatory regimes in biological systems and on a novel manifestation of such multi-rhythmicity.

In the case of bistability, when the steady state is plotted as a function of a control parameter, the curve takes a characteristic S or Z form, denoting the existence of a region in which three steady states coexist; generally, the steady states on the upper and lower branches are stable, while the middle state is unstable. Bistable behaviour is often associated with all-or-none transitions and hysteresis, the phenomenon by which the transition from a stable steady state *A* to a stable steady state *B* occurs at another value of the control parameter than the reverse transition from *B* to *A* [2,4]. In tristability, three stable steady states are separated by two unstable states. Many examples of the coexistence between two steady states have been discussed, mostly in theoretical models, in a wide range of fields extending from cell fate specification in developmental biology [6–11] to the dynamics of the cell cycle [12–17], and from immunology and other biological examples up to irreversible climatic transitions at a geophysical level (see [4], for a recent review). Tristability has been implicated in some cellular differentiation processes [18–22].

While examples of the coexistence between multiple steady states abound in biological systems, oscillatory behaviour appears to be even more common [4,23,24]. Biological rhythms are indeed encountered at all levels of biological organization, from metabolic oscillations in yeast to the circadian clock present in all eukaryotic organisms with a period close to 24 h, and from electrical oscillations in nerve and muscle cells to hormonal rhythms and predator–prey oscillations in ecology [25–31]. Sustained oscillations in nonlinear systems are generally of the limit cycle type: for a given set of parameter values, such oscillations keep the same amplitude and frequency regardless of initial conditions [32]. This property is responsible for the robustness of biological rhythms [28–30,33].

One reason why oscillations appear to be more widespread than multiple steady states is that the latter are more difficult to approach experimentally, given that a change in control parameters or in the initial conditions, due to a suprathreshold perturbation of the system, is required to demonstrate the coexistence between multiple stable steady states and the associated phenomenon of hysteresis. By contrast, oscillations are displayed as soon as the system moves into a domain of oscillatory behaviour in parameter space. A notable exception is when a stable steady state coexists with a stable oscillatory regime; the latter can be reached only when the system is moved sufficiently far away from the steady state, hence the name of *hard excitation* given to this situation [32], which has been observed experimentally in some electrically excitable cells [34].

The coexistence between multiple stable oscillatory states, i.e. multi-rhythmicity, represents the periodic counterpart of the coexistence between multiple stable steady states. Evidence for multi-rhythmicity has been obtained in a variety of theoretical models for regulated biochemical and cellular systems, as will be reviewed in §§2 and 3 below. The coexistence between two rhythmic patterns of electrical activity has also been found theoretically in studies of periodically stimulated cardiac cells [35–37] and in models of nerve cells regulated through mutual inhibition [38–40]. Besides these theoretical studies, experimental evidence for birhythmicity has been obtained in some chemical oscillatory systems [41,42] and also in a number of biological examples ranging from periodically stimulated cardiac cells [36,37] to nerve cells [43–45], the production of different registers of human voice [46,47], and an ecological system [48,49].

Besides hard excitation and the coexistence between two or three periodic regimes, models show that periodic oscillations may also coexist with a strange attractor corresponding to chaotic oscillations. The coexistence of two strange attractors has been found in some theoretical studies [5,50]. Multiple attractors were also observed in the case of spatial patterns, as exemplified by the coexistence of alternative rotor patterns in a chemical reaction displaying excitable and oscillatory behaviour [51].

Models are useful in that they bring to light the possible occurrence of multi-rhythmic behaviour. Indeed, as in the case of the coexistence between multiple stable steady states, the way to demonstrate multi-rhythmicity requires the prior knowledge that such phenomenon may exist. As in the case of bistability, to demonstrate the coexistence between multiple periodic attractors, one needs either to perturb the system, so as to elicit the transition to another stable attractor, or to vary continuously a control parameter back and forth in order to find evidence for hysteresis. The latter phenomenon reveals a region in which two stable attractors coexist.

The purpose of this article is twofold. First, we describe a variety of conditions in which the coexistence between two or three oscillatory regimes has been observed in a number of theoretical models for biochemical and cellular rhythms. Even though most experimental examples of multi-rhythmicity pertain, so far, to isolated neurons, neural circuits, or cardiac cells, it is in models for biochemical systems and cellular regulatory networks that the phenomenon has been most thoroughly and systematically investigated. We shall briefly examine models for multiply regulated enzyme reactions, cyclic AMP oscillations in *Dictyostelium* cells, cytosolic Ca^++^ oscillations, the *Drosophila* circadian clock, and the mammalian cell cycle. After addressing in §2 the endogenous mechanisms responsible for the onset of multi-rhythmicity in these models, we consider in §3 how multiple modes of entrainment can occur in the non-autonomous situation where an oscillator is subjected to forcing by an external periodic stimulus, or in the related autonomous situation where an oscillator such as the cell cycle is unidirectionally coupled to a second oscillator such as the circadian clock.

While we provide in §§2 and 3 a review of mechanisms that were previously implicated in the coexistence of multiple rhythms, we focus in §4 on a new type of mechanism for multi-rhythmicity based on the bidirectional coupling of two oscillatory systems. The study of a model for the bidirectional coupling of the cell cycle and the circadian clock recently showed that although the two oscillators generally synchronize in a unique manner, bidirectional coupling sometimes leads to a coexistence between 2 and 3 modes of synchronization characterized by different periods and waveforms [52]. Here we further document this phenomenon, which we propose to refer to as ‘multi-synchronization’. We show how the patterns of synchronization depend on the magnitude of the coupling strengths and on the time at which the coupling begins. In §5, to further characterize the coexistence of multiple rhythms we use the example of central pattern generators to distinguish multi-rhythmicity from the occurrence of different rhythms in different conditions, i.e. in distinct domains in parameter space. We conclude by addressing the physiological significance of multiple, coexisting biological rhythms.

## 2. Examples of endogenous multi-rhythmicity

To introduce multi-synchronization as a new source of multi-rhythmicity (see §4), it is useful to compare it to other mechanisms responsible for the coexistence of two or three stable periodic regimes. We shall briefly review how these alternative mechanisms were uncovered in models which were initially proposed to account for a unique regime of sustained oscillations observed experimentally in (i) yeast glycolysis, (ii) cyclic AMP (cAMP) signalling in *Dictyostelium* amoebae, (iii) Ca^++^ oscillations, (iv) the circadian clock and (v) the network of cyclin-dependent kinases (Cdks) driving the mammalian cell cycle. We shall focus on the mechanisms responsible for multi-rhythmicity in these oscillatory systems, and will relate them to other instances in which the phenomenon has been found.

### 2.1. Two instability-generating mechanisms coupled in series

The coexistence of two (birhythmicity) or three (trirhythmicity) stable periodic regimes was first observed in a model that represented an extension of a model previously proposed for glycolytic oscillations. These oscillations initially observed experimentally in the mid-1960s in suspensions of yeast cells and in yeast extracts, and subsequently in individual yeast cells, represent, to this day, the prototype of oscillatory behaviour in biochemical systems [23,24,53]. The experiments all point to the enzyme phosphofructokinase (PFK) as playing a major role in the mechanism of oscillations in this biochemical pathway. The mechanism involves the allosteric nature of the enzyme and its peculiar regulation, which involves its activation by a reaction product. The role of PFK was further corroborated by a recent analysis of glycolytic oscillations in intact yeast cells [54]. A two-variable allosteric model for the product-activated PFK reaction [55] accounts for many experimental observations, including the existence of a domain of oscillations bounded by two critical values of the substrate input rate in yeast extracts (see [23], for a detailed account).

The model for glycolytic oscillations describes the time evolution of the substrate and product in a product-activated allosteric enzyme reaction. It contains a single (positive) feedback loop, and, hence, a single instability-generating mechanism. While positive feedback is generally associated with bistability, it is coupled here to substrate consumption and to the removal of the reaction product. Both processes contribute to limit the explosive increase in product due to the autocatalytic regulation of the enzyme. The interplay of the positive and negative regulations underlies the oscillatory dynamics of the enzyme reaction. This model produces a single regime of simple, periodic oscillations, which match the experimental observations [23,55].

**Figure 1.**
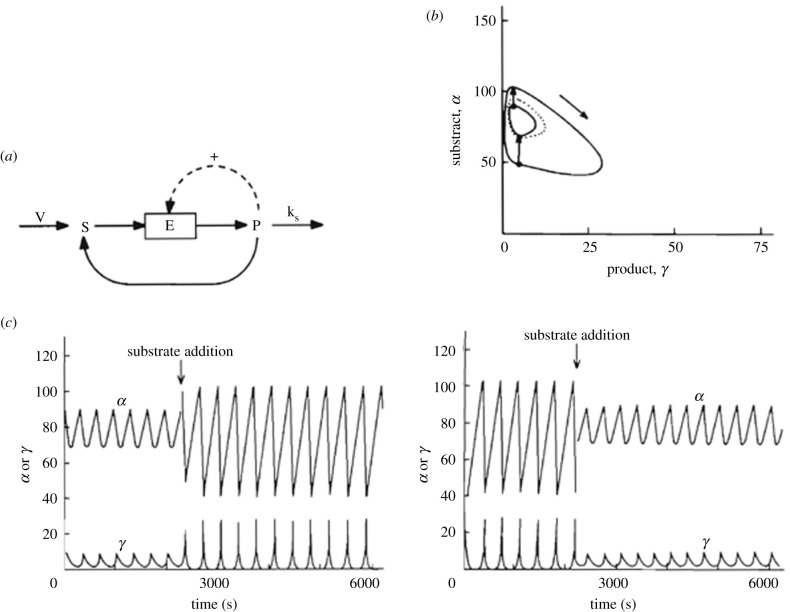
Birhythmicity in a two-variable model. (*a*) Scheme of the model of a product-activated enzyme with product P recycling into substrate S displaying birhythmicity [56]. The two coexisting limit cycles are shown in (*b*) with arrows indicating how to switch back and forth between the small-amplitude limit cycle and the large-amplitude limit cycle, upon addition of a pulse of substrate at an appropriate phase. The dotted trajectory denotes an unstable limit cycle separating the two stable limit cycles (solid trajectories). In (*c*) are shown the time series of the substrate and product concentrations, which are the two variables in the model schematized in (*a*). The time evolution corresponds to the switch indicated in (*b*), from low-amplitude to large-amplitude oscillations (left), and to the reverse switch (right). In both cases, switching is triggered by a suprathreshold pulse of substrate [56]. As shown in figure 2*b*, a similar switching between three stable modes of oscillations has been observed in the three-variable model of a multiply regulated biochemical system schematized in figure 2*a*.

This two-variable model was later modified to explore the onset of birhythmicity [56]. Using a one-dimensional bifurcation diagram in which the domain of sustained oscillations is determined as a function of the substrate input rate, we added a reaction of product recycling into substrate (see scheme in figure 1*a*) to create, within the oscillatory domain, a domain in which, over a range of values of the control parameter, a stable steady state coexists with large-amplitude oscillations. The stable steady state is then separated from the stable oscillatory regime by an unstable periodic regime, a situation referred to as hard excitation [32]. It is at the extremities of the island of stability of the steady state that a stable regime of small-amplitude oscillations is created, which coexist with the large-amplitude oscillations [56]. In the phase plane of the two-variable system, the two stable regimes of sustained oscillations correspond to a small-amplitude limit cycle embedded within a large-amplitude limit cycle; the two stable limit cycles are separated by an unstable periodic orbit (figure 1*b*). When applied at the right phases (figure 1*b*,*c*), perturbations in the form of additions of a pulse of substrate allow the system to switch reversibly between the two modes of stable, sustained oscillations, which differ by both the amplitude and frequency.

**Figure 2.**
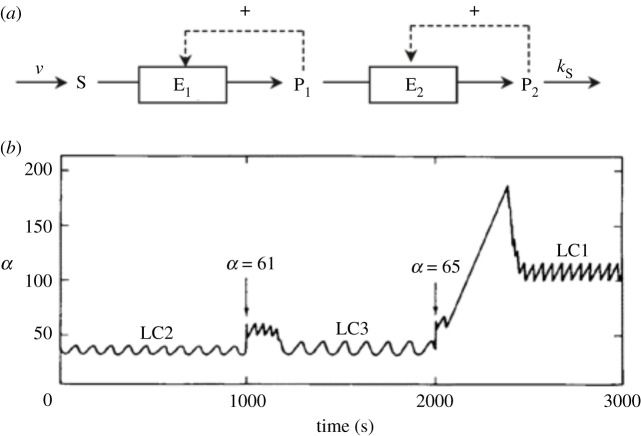
(*a*) Scheme of a three-variable model for two autocatalytic enzyme reactions coupled in series. This model [5] admits a large repertoire of oscillatory behaviour, including simple periodic oscillations, complex periodic oscillations (bursting), aperiodic oscillations (chaos), hard excitation (coexistence of a stable steady state and a stable limit cycle), birhythmicity and trirhythmicity. (*b*) In conditions of trirhythmicity, three stable limit cycles (LC1, LC2 and LC3) coexist. The curve shows how pulses of substrate (*α*) delivered at appropriate phases elicit the switch between the different regimes of sustained oscillations, associated with the transitions from LC2 to LC3, and from LC3 to LC1 [23,57,58].

The study of the model of figure 1*a* was based on the results previously obtained in a three-variable biochemical model developed to investigate complex patterns of oscillations, including chaotic behaviour. To this end, the model based on the role of PFK in glycolytic oscillations was extended by incorporating a second instability-generating mechanism in the form of a second positive feedback loop. The resulting three-variable model, schematized in figure 2*a*, thus represents two autocatalytic enzyme reactions coupled in series [5]. The repertoire of dynamic behaviour arising from the interplay between the two instability mechanisms is greatly enriched: simple periodic oscillations still represent the most common type of nonlinear behaviour, but additional phenomena were uncovered, such as complex periodic oscillations in the form of bursting, chaotic oscillations, and birhythmicity [5]. All these phenomena occur when two domains of instability are brought close to each other in parameter space until they overlap. Then the two instability-generating mechanisms are active at the same time, and complex oscillatory phenomena arise. When two domains of birhythmicity were found as a function of a control parameter, changing another parameter brought the two birhythmicity domains to overlap. In these conditions, three stable periodic regimes separated by two unstable oscillatory states were found (figure 2*b*). Such a situation corresponds to trirhythmicity [23,57,58]. As in figure 1*c*, transitions between the coexisting periodic regimes can be elicited by additions of pulses of substrate applied at the appropriate phases.

The model based on coupling in series two instability-generating mechanisms was built purposedly to explore the occurrence of complex oscillations, including chaos, in regulated biochemical systems. Multi-rhythmicity was discovered in this model by sheer serendipity: for certain parameter values, the system was found to evolve to distinct regimes of sustained oscillations when starting from different initial conditions. Birhythmicity (but not bursting or chaos) was found subsequently by following a similar scenario, in the two-variable extension of the model for glycolytic oscillations (figure 1). By contrast, the models described hereafter were developed to account for simple periodic behaviour observed experimentally. Multi-rhythmicity was found in these models unexpectedly or through a search in parameter space for regions where two domains of instability overlap [59].

### 2.2. Two paths coupled in parallel for cyclic AMP oscillations in *Dictyostelium* cells

The cAMP signalling system that controls aggregation of the cellular slime mould *Dictyostelium discoideum* represents a prototype for pulsatile intercellular communication [23]. Upon switching from the unicellular to the multicellular stage of their life cycle after being subjected to starvation, these amoebae aggregate by a chemotactic response to cAMP signals emitted with a periodicity of several minutes by cells behaving as aggregation centers. The mechanism that underlies the periodic generation of cAMP signals again relies on a positive feedback loop: cells produce cAMP which is synthesized intracellularly by the enzyme adenylate cyclase; cAMP is released into the extracellular medium where it binds to a cAMP receptor and thereby triggers the activation of adenylate cyclase, which transforms ATP in to cAMP. The resulting positive feedback loop in cAMP synthesis is counteracted by two limiting processes that involve, respectively, cAMP-induced receptor desensitization and cAMP hydrolysis by intracellular and extracellular forms of the enzyme phosphodiesterase.

The analysis of a model for cAMP signalling based on receptor desensitization accounts for the pulsatile synthesis and release of cAMP signals by *Dictyostelium* cells after starvation [60]. Somewhat surprisingly the model predicts the occurrence of more complex oscillatory phenomena in the form of bursting, chaos and birhythmicity [61,62]. Here again, the latter phenomenon is revealed by numerical simulations, which show that, depending on initial conditions, the signalling system is capable of evolving to two distinct modes of cAMP oscillations characterized by different waveforms and periods.

What is the origin of birhythmicity in the model for cAMP signalling, which contains but a single positive feedback loop? Oscillations can follow two paths in this model, depending on the process responsible for the decrease in cAMP synthesis after a peak in cAMP. Either the dominant process is cAMP receptor desensitization, which is accompanied by a decrease in the activation of adenylate cyclase and, hence, by a decrease in the rate of cAMP synthesis, or the dominant process is ATP consumption coupled to cAMP hydrolysis by phosphodiesterase. It appears that birhythmicity occurs when these limiting processes acquire comparable strength in contributing to the decrease in cAMP after a peak. Thus, the coexistence between two stable periodic regimes originates here from the operation of two parallel paths sharing the same positive feedback loop but differing by the process limiting the increase in cAMP.

### 2.3. Self-modulation of an oscillating system: the case of Ca^++^ oscillations

Birhythmicity was also observed by means of numerical simulations in a model for cytosolic Ca^++^ oscillations in which one of the parameters controlling the oscillations is modulated by one of the oscillating variables [63]. When an external signal such as a hormone or a neurotransmitter stimulates a cell, the signal triggers the synthesis of inositol trisphosphate (InsP_3_) which elicits the release of Ca^++^ from intracellular stores, in a process stimulated by cytosolic Ca^++^. This self-amplified process of Ca^++^-induced Ca^++^ release plays a primary role in the instability that leads to Ca^++^ oscillations.

The degradation of InsP_3_ occurs in two parallel ways, one of which is independent of Ca^++^ while the other is activated by Ca^++^. The activation of InsP_3_ degradation by cytosolic Ca^++^ introduces in the model a self-modulation of the InsP_3_ stimulatory signal that controls the oscillations. The repertoire of dynamic behaviour becomes much richer when this additional regulation is incorporated into the model for Ca^++^ oscillations [63]. In addition to periodic oscillations, bursting and chaos, the extended model predicts the occurrence of birhythmicity. The phenomenon is associated with the existence of two parallel paths for InsP_3_ degradation and, again, with the interplay of several regulatory feedback loops in a model producing sustained oscillations. Peculiar here is the fact that the input that controls the oscillations is controlled by the oscillatory output.

### 2.4. A feedback loop involving two branches in a model for the circadian clock

A related mechanism for the origin of complex oscillatory phenomena was found, again unexpectedly, in a model for the *Drosophila* circadian clock. The circadian clock represents the prototype of biological rhythms. These oscillations, which occur spontaneously in all eukaryotic cells and some bacterial species with a period close to 24 h, are controlled by the light–dark (LD) cycle that characterizes our environment. Because of the control by the LD cycle, the circadian clock represents a major example of biological rhythm naturally subjected to periodic forcing. We will return to the effect of such forcing in §3 below.

In *Drosophila*, which is one of the most studied organisms in regard to the molecular mechanism of the circadian clock, the periodic synthesis of clock proteins such as PER and TIM originates from a negative feedback exerted by the PER-TIM complex on the expression of the *Per* and *Tim* genes. This negative feedback on transcription was later shown to proceed via the inhibition of a complex formed by two activators that induce the expression of *Per* and *Tim*. A first model for the *Drosophila* circadian clock, based on a negative feedback exerted by PER on the transcription of its gene, accounted for sustained oscillations of the limit cycle type [30]. In this model, a single regime of oscillations was found.

**Figure 3.**
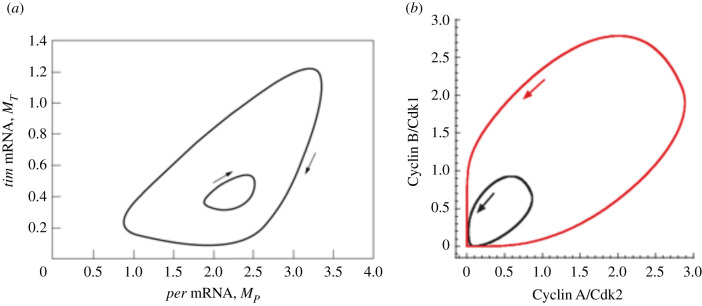
Coexistence of two stable limit cycles (birhythmicity) in (*a*) a model for the *Drosophila* circadian clock [65] and (*b*) a skeletal model for the mammalian cell cycle [66]. The curves represent projections onto a two-variable phase plane of the trajectory of a 10-variable system (*a*) and of a 5-variable system (*b*).

The subsequent discovery of the role of the TIM protein led to the construction of a second model for the *Drosophila* circadian clock incorporating TIM, which forms with PER the complex that inhibits the transcription of the *Per* and *Tim* genes [64]. Containing ten variables (twice as many as the negative feedback model based on PER alone), but still a single negative feedback loop exerted by the PER-TIM complex, this model also accounts for the occurrence of circadian oscillations in the levels of the two proteins and their mRNAs. Unexpectedly, complex oscillatory phenomena were uncovered in the PER-TIM model in the form of birhythmicity (figure 3*a*) and chaos [65]. Such dynamic phenomena occurred in the presence of asymmetries in the PER and TIM branches leading to the formation of the PER-TIM complex that plays the key role in the origin of oscillations. The levels of PER and TIM are governed by the rates of synthesis and degradation of the two proteins and of their mRNAs. Any difference in any of these rates can lead to a ‘dynamic imbalance’ in the formation of the regulatory complex. Numerical simulations indicate that birhythmicity, as well as period-doubling bifurcations leading to chaos, occurred only in the presence of such ‘dynamic imbalance’ between the PER and TIM branches involved in the negative feedback loop.

### 2.5. Multiple oscillatory circuits in a model for the mammalian cell cycle

Let us finally turn to yet another source of endogenous multi-rhythmicity in a regulated biochemical network. This time, the mechanism relies on the interplay of a multiplicity of oscillatory circuits in a complex network that governs the dynamics of the mammalian cell cycle.

In mammalian cells, a network of Cdks controls the progression along the G1, S, G2 and M phases of the cell cycle. The model proposed for the mammalian cell cycle [67] contains a large number of variables and takes into account multiple layers of biochemical regulation which bear, respectively, on cyclin synthesis and degradation, Cdk regulation through reversible phosphorylation and binding of Cdk inhibitors. The model predicts the occurrence of sustained oscillations in the various cyclin–Cdk complexes. The ordered activation of these complexes brings about the transitions between the successive phases of the cell cycle. The passage from a stable steady state in the Cdk network—corresponding to cell cycle arrest—to Cdk oscillations occurs through a bifurcation that is controlled by a variety of factors such as the levels of growth factors, Cdk inhibitors and oncogene products, and by the extracellular matrix and cell density [67,68].

Although a single oscillatory regime is generally observed in the model for the Cdk network, at least four oscillatory sub-circuits can be isolated within it. Each of these four circuits, if isolated artificially, can oscillate on its own. However, in physiological conditions, the four oscillatory circuits, far from being isolated, are tightly coupled and oscillate in concert with the same period. This ‘internal synchronization’ results in the existence of a single oscillatory regime, i.e. mono-rhythmicity, in the Cdk network [67]. By contrast, when parameter values are altered so that the internal coupling between the oscillatory circuits becomes weaker, complex oscillations in the form of bursting or chaos can occur [69]. This situation stems from that several sub-circuits are now capable of expressing their own oscillatory potential while remaining, nevertheless, linked to the other parts of the network. Complex oscillatory phenomena arise from the interplay of the different oscillatory circuits which fail to synchronize internally. Similar results, as well as birhythmicity (figure 3*b*), were observed in a reduced model containing only five variables, that retains the same regulatory structure as the full model for the Cdk network [66].

## 3. Multi-rhythmicity due to periodic forcing of an oscillatory system or to unidirectional coupling of two oscillators

### 3.1. Forcing of an oscillatory system by an exogenous periodic stimulus

The examples of multi-rhythmicity discussed so far belong to the class of autonomous systems not subjected to any forcing by an external oscillatory variation in some control parameter. As mentioned above, the circadian clock is unique in providing an example of a key biological rhythm driven by the periodic variation of the environment. We studied the effect of periodic forcing of the circadian clock by the LD cycle in a simple three-variable model for the circadian clock, based on negative auto-regulation of gene expression. The effect of the LD cycle was taken into account by specifying that the rate of expression of the clock gene varied periodically, increasing during the light phase and decreasing in the dark phase, as observed in the fungus *Neurospora* and in mammals (by contrast, in *Drosophila*, the effect of light is to increase the rate of degradation of the TIM protein).

Depending on parameter values and on the forcing period, the forcing of the circadian clock model by the LD cycle was found to elicit entrainment by the LD cycle, quasi-periodic oscillations, or chaos [70]. Entrainment was the most common type of behaviour produced by forcing. No evidence for multi-rhythmicity was obtained in these conditions, but this does not exclude the occurrence of the phenomenon, since the results were obtained by numerical integration of the differential equations that govern the circadian clock model, and that the domains of multi-rhythmicity are often reduced in size in parameter space, compared to the domains of entrainment or complex oscillations.

Another example of a similar situation was studied experimentally, namely, the entrainment of oscillations in the NFKB signalling system by pulsatile stimulation by TNF [71]. Theoretical studies of this system later provided evidence for multi-rhythmicity in the form of coexistence between different patterns of entrainment [72,73]. Each domain of entrainment takes the characteristic form of an Arnold tongue, which becomes progressively wider as the coupling strength increases. The domain of birhythmicity corresponds to the region where different Arnold tongues, associated with distinct patterns of entrainment, overlap. In the presence of fluctuations, transitions between different modes of entrainment were shown to be induced by noise, a phenomenon referred to as ‘mode hopping’ [72]. From a functional point of view, the interest of the phenomenon stems from that different modes of NFKB oscillations appear to induce different patterns of gene expression [71–74].

Pulsatile stimulation also occurs naturally in the heart, which contains different pacemaker tissues behaving as autonomous oscillators, The stimulation of cardiac tissue by externally applied current pulses has long provided an important approach to the experimental study of complex heart rhythms [75]. This approach, complemented by theoretical studies, was successfully applied to demonstrate a variety of nonlinear phenomena such as period-doubling oscillations and chaos in periodically stimulated cardiac cells [76]. Guevara *et al.* [36] obtained experimental evidence of birhythmicity by studying the response to current pulses in aggregates of embryonic chick ventricular cells. They observed the coexistence between two patterns of entrainment of these cells by current pulses, with hysteresis between the 1 : 1 and 2 : 1 entrainment modes, i.e. one cardiac action potential is elicited by every pulse or every second pulse, respectively (see fig. 23.5 in [36]). Similar results on the coexistence of these two entrainment modes were also obtained in experiments on single rabbit ventricular cells and accounted for by numerical simulations based on a model for ventricular cells [37]. These observations were in agreement with the predictions of a theoretical study of periodic forcing of an oscillatory system [35], which showed an overlap of Arnold tongues corresponding to different modes of entrainment.

The effect of pulsatile stimulation of an oscillatory system has also been studied in regard to metabolic oscillations. Thus, the experimental and theoretical study of the forcing of glycolytic oscillations in yeast extracts by a periodic input of substrate provided evidence for entrainment and subharmonic entrainment to twice or three times the input period [26]. Neither multi-rhythmicity nor chaos was observed in these studies, but the duration of the recordings was much more limited in this system than in the experiments on cardiac cells, owing to the period of glycolytic oscillations, which is of the order of minutes in yeast extracts.

### 3.2. Unidirectional coupling of two oscillators

**Figure 4.**
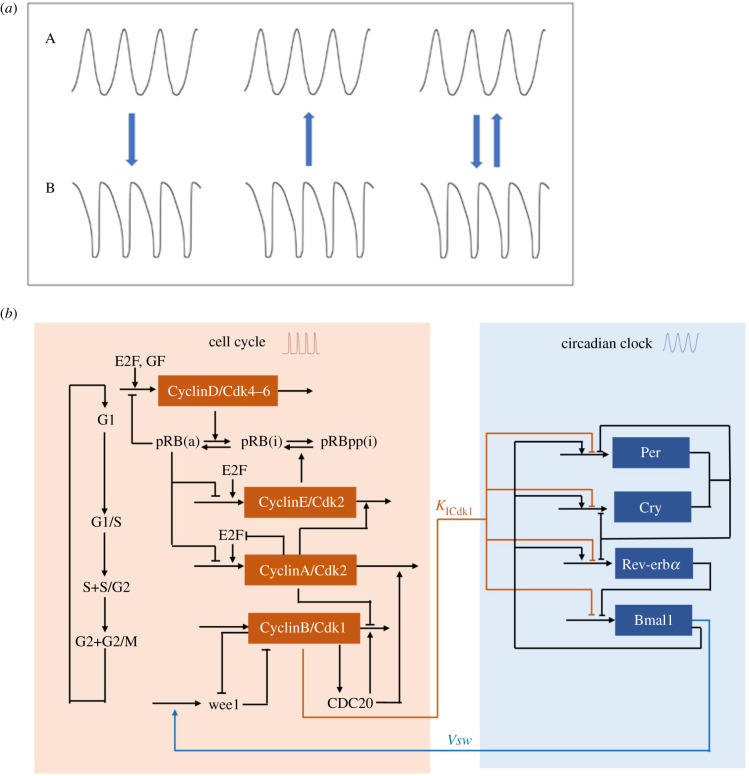
(*a*) Schemes showing two modes of unidirectional coupling versus bidirectional coupling of two distinct oscillators. (Left) Oscillator A as input driving oscillator B. (Centre) Oscillator B as input driving oscillator A. (Right) Bidirectional coupling of oscillators A and B. Before coupling, the two oscillators generally differ by their period. In one realization of such coupling considered in §§3 and 4, oscillators A and B refer, respectively, to the circadian clock and to the cell cycle in mammalian cells. (*b*) Scheme showing the bidirectional coupling of the Cdk network (left) and the circadian clock (right) considered in §4. The two networks display sustained oscillatory behaviour of the limit cycle type, and their coupling can take multiple forms which generally bring the two networks to oscillate at a unique synchronization period [52]. In conditions of bidirectional coupling that lead to multi-rhythmicity in the form of multi-synchronization (see §4), the circadian clock controls the cell cycle by inducing, with a maximum rate *v*_sw_, the expression of the kinase Wee1 that inhibits Cdk1, while the cell cycle kinase Cdk1 inhibits the circadian clock by repressing the expression of the circadian clock genes, with an inhibition constant denoted *K*_ICdk1_. The model for the bidirectional coupling of the two oscillatory networks is governed by a set of some 62 differential equations, which are listed in the supporting information in our previous publication [77], where the computer code used in numerical simulations can also be found.

Multi-rhythmicity can also occur through the coupling an oscillatory system to another oscillator. As schematized in figure 4*a*, when two oscillators A and B interact, three cases can be considered. Either oscillator B is forced by oscillator A (left panel) or A is forced by B (middle panel); these two situations correspond to the unidirectional coupling of A and B. The third case of bidirectional coupling (right panel) will be considered in §4 below. The unidirectional coupling of two oscillators was considered early on by Tyson [78] who investigated the coupling in series of two ‘Brusselator’ models admitting limit cycle oscillations. This study provided evidence for complex periodic or quasi-periodic oscillations.

In regard to biological systems, the links between the cell cycle and the circadian clock allow us to investigate the coupling of two cellular oscillators [79–83]. Experimental evidence indicates that the Cdk network that controls the dynamics of the mammalian cell cycle (oscillator B) is regulated in several ways by the circadian clock (oscillator A), e.g. through the control of Wee1—a kinase that inhibits Cdk1—by BMAL1, a key circadian regulator [84]. The effect of such unidirectional coupling was determined by linking the detailed molecular models for the circadian clock and for the cell cycle in mammalian cells [79,85]. The detailed numerical analysis of the model for the coupled system showed that entrainment of the cell cycle by the circadian clock of 24 h period occurs over a large range of values of the autonomous period of the cell cycle. The domains of entrainment were established as a function of the coupling strength. Outside the domains of entrainment, complex oscillations or chaos were found [79]. Further numerical study of the unidirectional coupling of the cell cycle to the circadian clock revealed the possibility of multiple modes of entrainment. Thus, two or three different types of entrainment sometimes coexist in the same conditions, i.e. for the same set of parameter values [85]. The evolution towards either one of the stable modes of entrainment depends on initial conditions. These results are supported by experimental [80] and numerical evidence [86] for the coexistence between different patterns of entrainment of the cell cycle by the circadian clock in mammalian cells.

## 4. Multi-synchronization in the bidirectional coupling of two oscillators

Let us now turn to a mechanism of multi-rhythmicity that differs from the various mechanisms considered so far for the coexistence between two or three regimes of sustained oscillations. This mechanism is closely related to the synchronization of self-sustained oscillators, which has become a major field of research in nonlinear science. Most models for synchronization in networks of biological oscillators are of an abstract mathematical nature, which makes them amenable to analytical study [28,87–89]. Here, we approach this problem by means of numerical simulations of a detailed molecular model for the bidirectional coupling of two major cellular rhythms.

### 4.1. Bidirectional coupling of two oscillators as a source of multi-rhythmicity

Rather than being unidirectional, which case was addressed in §3.2, it appears that the coupling between the cell cycle and the circadian clock is bidirectional (as schematized in figure 4*a*, right panel) and that the relative magnitude of the control exerted by each oscillator on the other depends on cell type and on experimental conditions [80]. Several components of the Cdk network that controls the dynamics of the mammalian cell cycle (see §2.5)—e.g. the cyclin-dependent kinase Cdk1 involved in triggering mitosis—indeed regulate some components of the circadian clock, while components of the latter—particularly transcription factors such as BMAL1—control the expression of some cell cycle genes [82,84,90–96]. New links between the two networks continue to be uncovered [83,97]. The dynamical consequences of such bidirectional coupling were explored numerically in a computational model based on detailed models for the mammalian circadian clock and the mammalian cell cycle [52]. Various forms of bidirectional coupling based on experimental observations were considered, including the inhibition of transcription in the M phase [98]. The latter mode of regulation was previously shown to lead, on its own, to a single mode of entrainment in the case of unidirectional coupling of the circadian clock to the cell cycle [52,99].

A major conclusion from this numerical study is that bidirectional coupling enhances the robust synchronization of the cell cycle and the circadian clock [52]. Over a large range of coupling strengths, the two networks synchronize at a unique period, which is close to, or between, the autonomous periods of the two oscillators prior to their coupling. Sometimes the synchronization period lies outside the range defined by the two autonomous periods. By contrast to the results obtained in conditions of unidirectional coupling of the cell cycle to the circadian clock [79,85], when the coupling becomes bidirectional the two oscillators tend to synchronize in the form of simple periodic oscillations rather than exhibiting complex oscillatory behaviour or chaos [52].

Preliminary evidence showed that the bidirectional coupling of the models for the cell cycle and the circadian clock can provide a new source of multi-rhythmicity in the form of multiple modes of synchronization of the two oscillatory networks. In some conditions, and for certain types of coupling, instead of synchronizing in a single mode of simple periodic oscillations, the two systems may synchronize in two (fig. 9 in [52]) or even three distinct types of simple periodic oscillations (see electronic supplementary material, fig. S7, in [77]), which are stable in the same set of conditions. Here we further investigate this phenomenon of multi-synchronization and focus on the situation in which the phenomenon was observed numerically, namely, the coupling of the cell cycle to the circadian clock occurs via BMAL1 induction of the kinase Wee1, while the coupling of the circadian clock to the cell cycle occurs via mitotic repression of transcription, controlled by Cdk1 (see a simplified scheme of the bidirectionally coupled cell cycle and circadian networks in figure 4*b*). Further details and a list of the evolution equations in these conditions are given in §§2 and 5 in the supplementary material of our previous publication [77].

### 4.2. Multi-synchronization: bifurcations, time series and phase space trajectories

**Figure 5.**
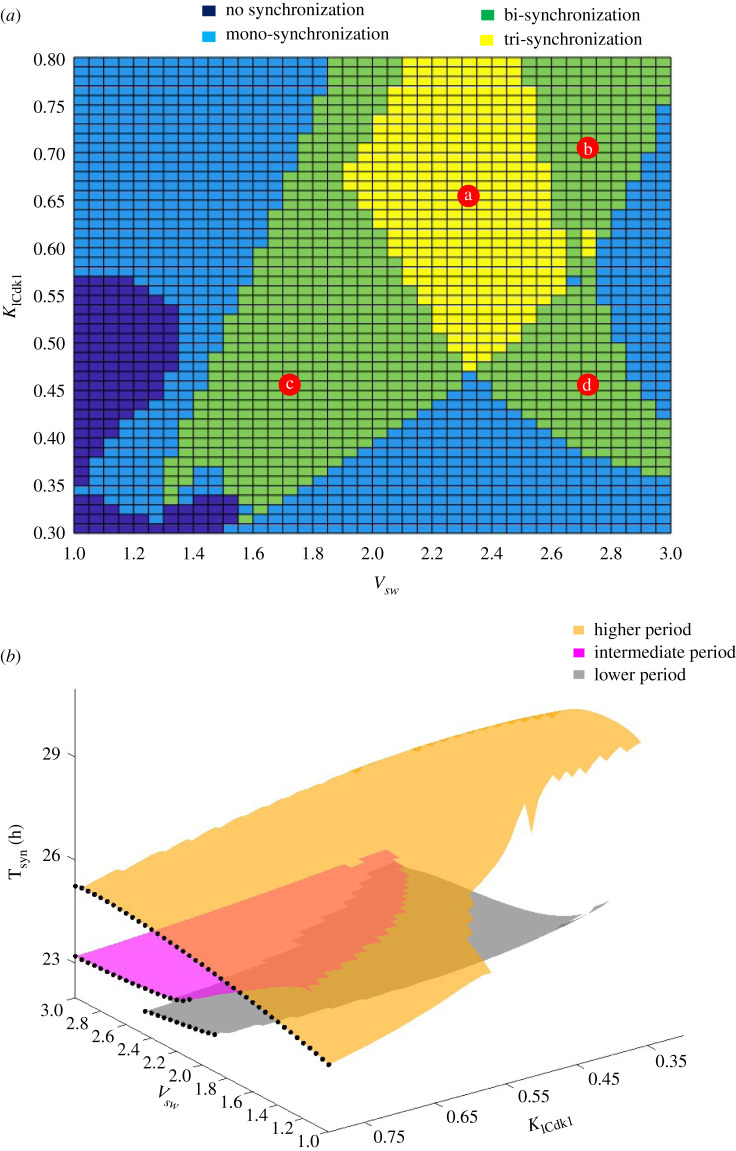
Bifurcation diagram for multi-synchronization. The diagram shows the domains of coexistence of 2 or 3 stable modes of synchronization of the circadian clock and of the cell cycle, in conditions of bidirectional coupling in a model for the coupling of the two oscillators schematized in figure 4*b*. (*a*) The bifurcation diagram is established as a function of parameters *v*_sw_ (in µM h^−1^) and *K*_ICdk1_ (in µM), which measure, respectively, the strength of coupling the cell cycle to the circadian clock and the strength of coupling the circadian clock to the cell cycle (for further details on the form of the bidirectional coupling and on the evolution equations for the coupled cell cycle–circadian clock model, see [77]). The autonomous period of the cell cycle and the circadian clock before coupling is 19.1 h and 24 h, respectively. The scaling parameter *eps* used to fix the cell cycle period in the simulations is equal to 22.5 [77]. The lighter blue region and the darker blue region correspond, respectively, to a unique mode of synchronization and to the failure of synchronization of the two oscillators. The green and yellow regions correspond to multi-synchronization: in the two green regions, two modes of stable synchronization of the two oscillators are observed (as illustrated in figure 7), while in the yellow region in which the two green regions overlap, three distinct modes of synchronization of the two oscillators are observed (as illustrated in figure 6). (*b*) Three-dimensional plot showing the period of synchronization as a function of *v*_sw_ and *K*_ICdk1_, in the conditions of (*a*). Multi-rhythmicity arises from the overlap of two or three distinct surfaces, which corresponds to a relatively higher (yellow), intermediate (magenta) or lower (grey) synchronization period. The black dots, corresponding to the value *K*_ICdk1_ = 0.8 µM, are plotted to help visualize the relative positions of the three surfaces. Upon increasing *v*_sw_ from the lowest to the highest value, we successively observe regions of mono-rhythmicity (high synchronization period), birhythmicity (coexistence of high and low synchronization periods), trirhythmicity (coexistence of high, intermediate and low synchronization periods), and another region of birhythmicity (coexistence of high and intermediate synchronization periods), Outside these regions of overlap we observe a single mode of synchronization, as illustrated in electronic supplementary material, figure S2.

We show in figure 5*a* a bifurcation diagram established as a function of two parameters, *v*_sw_ and *K*_ICdk1_, which measure, respectively, the strength of coupling of the cell cycle to the circadian clock via BMAL1 induction of the kinase Wee1 (see §2 in [77]), and the strength of coupling of the circadian clock to the cell cycle via mitotic repression of transcription, controlled by Cdk1 (see §5 in [77]). The diagram indicates the existence of four regions in this parameter space: a region where the two networks synchronize in a unique way (mono-synchronization, in light blue), a region in which the two oscillators may synchronize in two distinct ways (bi-synchronization, in green), and a region in which the two oscillators may synchronize in three distinct ways (tri-synchronization, in yellow). Finally, in another region adjacent to the region of mono-synchronization, at relatively low coupling strengths, the two oscillators fail to synchronize (dark blue region in figure 5*a*).

In figure 5*b* we plot the synchronization period as a function of the same parameters, *v*_sw_ and *K*_ICdk1_. We see that multi-rhythmicity arises from the overlap of two or three distinct surfaces, which correspond to a relatively higher (yellow), intermediate (magenta) or lower (grey) synchronization period (see legend to figure 5*b*). The overlapping portions of two or three of these surfaces thus correspond to the coexistence of two or three distinct modes of synchronization. Outside the regions of overlap, we observe a single mode of synchronization or the absence of synchronization (in the region in white).

**Figure 6.**
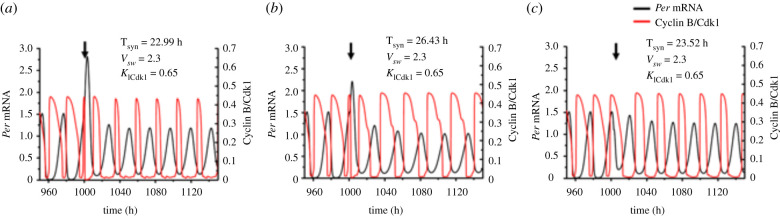
Coexistence of three distinct modes of synchronization upon bidirectional coupling of the cell cycle and the circadian clock. The time series show the 3 modes of synchronization observed in conditions corresponding to point *a* in figure 5*a*, where *v*_sw_ = 2.3 µM h^−1^, *K*_ICdk1_ = 0.65 µM. Shown are the time evolution of a cell cycle variable (Cyclin B/Cdk1) and of a circadian variable (*Per* mRNA). To demonstrate numerically the existence of the different modes of synchronization, we modify the time (marked by a vertical arrow) at which the bidirectional coupling begins, as explained in the text and in the legend to figure 9. (*a*) When the cell cycle and the circadian clock are bi directionally coupled at 1000 h, the two oscillatory networks synchronize at a period equal to 22.99 h. (*b*) When the bidirectional coupling occurs at 1001 h, the synchronization period is 26.43 h. (*c*) When the bidirectional coupling occurs at 1003 h, the oscillations synchronize at a period equal to 23.52 h. For details on the model for the coupled circadian clock and cell cycle oscillators, see [52] and the electronic supplementary material information therein [77].

An example of trirhythmicity corresponding to point *a* in the diagram of figure 5*a* is shown in figure 6, where the time course of one circadian variable (*Per* mRNA) and one cell cycle variable (Cyclin B/Cdk1) are plotted. We observe that in the same conditions, i.e. for the same set of parameter values, upon bidirectional coupling the two oscillators can synchronize in three ways with a period equal to 22.99 h (figure 6*a*), 26.43 h (figure 6*b*) or 23.52 h (figure 6*c*). The three stable periodic regimes are characterized by markedly different amplitudes and waveforms.

**Figure 7.**
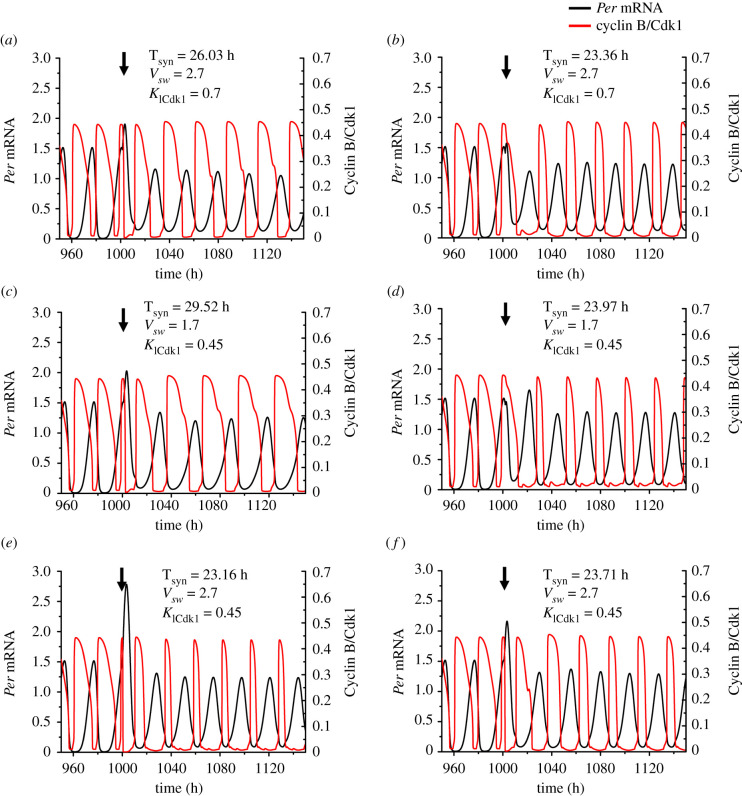
Three examples illustrating the coexistence of two distinct modes of synchronization upon bidirectional coupling of the cell cycle and the circadian clock. The time series show the coexistence of 2 modes of synchronization in conditions corresponding to the points marked *b*, *c*, *d* in figure 5*a*. The vertical arrow indicates the time at which the two oscillators are bidirectionally coupled. For the upper row, corresponding to point *b* in figure 5*a*, *v*_sw_ = 2.7 µM h^−1^, *K*_I__Cdk1_ = 0.7 µM. The cell cycle and circadian clock can be synchronized to 26.03 h (*a*) or 23.36 h (*b*) when they are bidirectionally coupled at 1001.5 h or at 1002 h, respectively. For the middle row corresponding to point *c* in figure 5*a* where *v*_sw_ = 1.7 µM h^−1^, *K*_ICdk1_ = 0.45 µM, the two oscillatory networks synchronize at a period of 29.52 h (*c*) or 23.97 h (*d*) when they are coupled at 1001 h or 1002 h, respectively. For the bottom row corresponding to point *d* in figure 5*a* where *v*_sw_ = 2.7 µM h^−1^, *K*_ICdk1_ = 0.45 µM, the two oscillatory networks synchronize at a period of 23.16 h (*e*) or 23.71 h (*f*) when their bidirectional coupling begins at 1000 h or 1001 h, respectively. For details on the model for the coupled circadian clock and cell cycle oscillators in mammalian cells, see [52] and supporting information therein [77].

Three examples of birhythmicity are represented in figure 7, where figures 7*a*,*b*, 7*c*,*d* and 7*e*,*f* correspond to points *b*, *c* and *d* in the diagram of figure 5*a*, respectively. These points were selected so as to illustrate the coexistence between two modes of synchronization in the different birhythmicity regions in the bifurcation diagram in figure 5*a*.

Let us stress again that the most common situation observed in the model is that in which the cell cycle and the circadian clock synchronize in a unique way for a given set of parameter values. This phenomenon of mono-synchronization was studied in detail in our previous publication [52]. For the sake of completeness we show in electronic supplementary material, figure S1, the diagram of figure 5 in which we marked the points *e*, *f*, *g* corresponding to three examples of mono-rhythmicity. Shown in electronic supplementary material, figure S2, are the corresponding time series showing the evolution towards a single mode of synchronization after the onset of bidirectional coupling. The three mono-rhythmic situations selected correspond to the three surfaces shown in figure 5*b*, in regions where they do not overlap. Thus the two oscillators synchronize at a period of 23.64 h (A, for a point *e* corresponding to the grey surface in figure 5*b*, with relatively lower synchronization periods), 30.27 h (B, for point *f* corresponding to the yellow surface in figure 5*b*, with relatively larger synchronization periods), or 23.47 h (C, for point *g* corresponding to the magenta surface in figure 5*b*, with intermediate synchronization periods).

**Figure 8.**
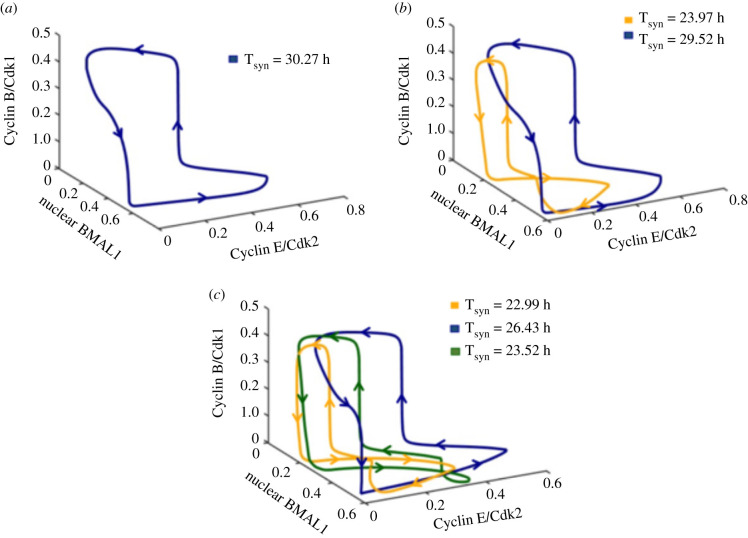
Phase space trajectories showing a single mode of synchronization or the coexistence of 2 or 3 modes of synchronization upon bidirectional coupling of the cell cycle and the circadian clock. The asymptotic trajectories followed in phase space by the bidirectionally coupled cell cycle–circadian clock model are projected as a function of two cell cycle variables (Cyclin B/Cdk1, Cyclin E/Cdk2) and one circadian variable (nuclear BMAL1). The arrows indicate the direction of movement on the closed trajectories. (*a*) Limit cycle corresponding to the oscillations shown in electronic supplementary material, figure S2B, when the bidirectional coupling leads to a unique mode of synchronization to 30.27 h for *v*_sw_ = 1.25 µM h^−1^, *K*_ICdk1_ = 0.4 µM. (*b*) Limit cycle corresponding to the oscillations in figure 7*c*,*d*, when *v*_sw_ = 1.7 µM h^−1^, *K*_ICdk1_ = 0.45 µM. In this case, the cell cycle and the circadian clock can synchronize at a period of 23.97 h (orange) or 29.52 h (dark blue). (*c*) Limit cycles corresponding to the oscillations in figure 6, when three different modes of synchronized oscillations occur, for *v*_sw_ = 2.3 µM h^−1^, *K*_ICdk1_ = 0.65 µM. The bidirectionally coupled system can evolve to synchronized oscillations with a period of 22.99 h (orange), 26.43 h (dark blue) or 23.52 h (dark green). For details on the model for the coupled circadian clock and cell cycle oscillators, see [77].

The phase space trajectories corresponding to a single mode of synchronization (mono-rhythmicity), and to two or three modes of synchronization are shown in figure 8*a–c*, respectively. The bidirectionally coupled cell cycle–circadian clock model contains some 62 variables [77]. The curves in figure 8 represent projections of the trajectories followed by the full system of differential equations listed in [77] onto a three-dimensional phase space formed by the concentrations of a circadian clock variable, nuclear BMAL1, and two cell cycle variables, Cyclin B/Cdk1 and Cyclin E/Cdk2. Because some of these trajectories appear to be close to each other, at least in their three-dimensional projections, it will be interesting to study the effect of noise in these instances of multi-rhythmicity.

### 4.3. The selected mode of synchronization depends on the time of the coupling

The different coexisting attractors, corresponding to distinct modes of synchronization, can be reached from different initial conditions. Each mode of synchronization possesses its basin of attraction and is characterized by its own period and waveform, as illustrated in figures 6 and 7 in the cases of trirhythmicity and birhythmicity, respectively. To illustrate the dependence on initial conditions we could change the initial conditions for one or more variables, as was done in figs 8 and 9 in [85] in the case where multi-rhythmicity arises from the unidirectional coupling of the cell cycle to the circadian clock. We use here an alternative procedure that yields similar results, by changing the time at which the bidirectional coupling starts when the cell cycle and circadian oscillators at first oscillate independently. Then, the change in the time at which the bidirectional coupling begins corresponds to a different set of initial conditions for all variables (rather than a single variable) of the coupled system.

**Figure 9.**
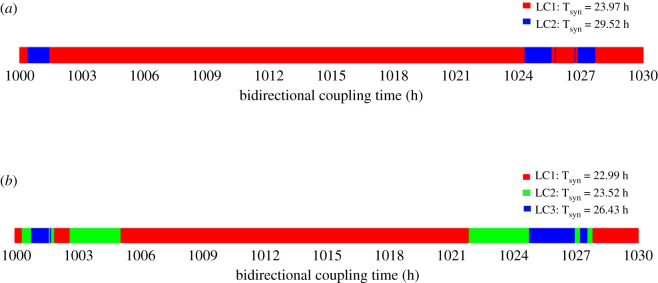
Selected mode of oscillations as a function of coupling time in conditions of multi-synchronization. When two (*a*) or three (*b*) modes of synchronization coexist in the bidirectionally coupled cell cycle–circadian clock model, the evolution towards one or the other limit cycle (LC) trajectories depends on the timing at which the bidirectional coupling begins. In both panels, the time at which the bidirectional coupling begins is changed from 1000 h to 1030 h by intervals of 0.05 h. (*a*) The cell cycle and the circadian clock can synchronize to 23.97 h (LC1, red), 29.52 h (LC2, blue) depending on the time at which bidirectional coupling begins. The birhythmicity corresponds to the case illustrated in figure 7*c*,*d*. (*b*) The cell cycle and the circadian clock can synchronize to 22.99 h (LC1, red), 23.52 h (LC2, green) and 26.43 h (LC3, blue), depending on the time at which bidirectional coupling is initiated. The trirhythmicity corresponds to the case illustrated by the time series in figure 6. The period of the cell cycle and the circadian clock prior to coupling is equal to 19.1 h and 24 h, respectively.

Shown in figure 9*a*,*b* is the dependence of the final mode of synchronization on the time at which the coupling begins, in the cases of birhythmicity and trirhythmicity, respectively. The time at which the coupling begins is increased progressively from 1000 h to 1030 h, by increments of 0.05 h. In figure 9*a* most coupling times in this interval lead to the selection of limit cycle 1 (LC1, in red), of synchronization period equal to 23.97 h. More rarely the coupling time leads to the evolution to limit cycle 2 (LC2, in blue) of synchronization period equal to 29.52 h. A similar procedure followed in the case of trirhythmicity (figure 9*b*) shows the alternation between limit cycles LC1 (in red, synchronization period equal to 22.99 h), LC2 (in green, synchronization period equal to 23.52 h) and LC3 (in blue, synchronization period equal to 26.43 h).

### 4.4. Complex periodic behaviour or chaos in the case of synchronization failure

What is the behaviour of the bidirectionally coupled system when the two oscillators fail to synchronize? We show in the upper panel of electronic supplementary material, figure S3, that complex periodic oscillations may occur, as in point *h* in the diagram of electronic supplementary material, figure S1. When the coupling strength is sufficiently low, as in point *i* in electronic supplementary material, figure S1, aperiodic oscillations may occur (electronic supplementary material, figure S3, lower panel). We previously reported the occurrence of these two types of complex oscillatory behaviour in the case of unidirectional coupling of the cell cycle to the circadian clock [79,85]. Let us stress again, however, that after the onset of bidirectional coupling, the evolution of the two oscillatory networks to a single regime of simple periodic oscillations characterized by a unique synchronization period remains by far the most common type of oscillatory behaviour in parameter space [52].

## 5. Discussion

The possible occurrence of multiple limit cycles has first been considered from a purely mathematical point of view, and the question of finding the maximum number of limit cycles in two-dimensional polynomial systems is known as the XVIth problem posed by Hilbert. The coexistence of multiple periodic solutions of the limit cycle type has subsequently been addressed theoretically in a variety of other fields, including physics, chemistry, biology and economics. A related line of research aims at developing strategies to suppress birhythmicity and restore mono-rhythmicity in controlled physical systems [100]. Here we focused on the mechanisms that underlie the coexistence of multiple periodic regimes in oscillatory biological systems and identified a new mechanism, which we refer to as multi-synchronization, for the occurrence of multi-rhythmicity.

### 5.1. Multi-rhythmicity: the periodic counterpart of bistabiliy and tristability

Multi-rhythmicity, which takes the form of a coexistence between two (birhythmicity) or three (trirhythmicity) stable regimes of sustained oscillations, represents the periodic counterpart of the coexistence between two (bistability) or three (tristability) stable steady states in biological, chemical or physical nonlinear systems. While theoretical and experimental evidence for multi-stability abounds, such evidence is more limited in the case of multi-rhythmicity. To compare the two situations, let us recall that bistability has been observed experimentally, and/or predicted theoretically, in a large variety of biological systems (see [4] for a recent review), most notably in the context of irreversible cell cycle transitions [12–17], genetic regulation [101], and cell fate specification in embryogenesis [6–11]. Tristability has also been implicated in various developmental processes [18–22].

The coexistence of multiple rhythms appears to be less widespread than the coexistence of multiple steady states. Some chemical examples of birhythmicity have been reported experimentally [41,42]. In biological systems, birhythmicity was demonstrated experimentally and theoretically in periodically stimulated cardiac cells, in which two patterns of entrainment may coexist [36,37]. Another example pertaining to the physiology of voice production by vocal fold vibrations has been studied theoretically and in excised larynx experiments. These studies demonstrated a region of coexistence of distinct vibration patterns corresponding to the chest and falsetto registers of human voice [46,47]. In neurobiology, the R15 neuron in *Aplysia* is able to switch spontaneously, or upon perturbation, between tonic and bursting oscillations [44]. Other neurons also display bistable firing behaviour [43]. Birhythmicity has been found in a number of theoretical models for neural circuits controlled by mutual inhibition [38,40].

Central pattern generators (CPGs) are neuronal networks that control rhythmic physiological functions, such as movements or respiration [102–104]. The lobster or crab somatogastric system represents one of the most studied CPGs. It is capable of displaying two different rhythms: a fast pyloric rhythm, and a slower gastric-mill rhythm [45]. Certain neurons take part in the generation of each of these rhythms [45,105–107]. Nested CPGs producing two distinct rhythms have also been characterized in the control of grooming in *Drosophila* [108]. These dual rhythmic properties of CPG neurons might possibly be interpreted in terms of multi-rhythmicity.

While rhythmic behaviour represents one of the most conspicuous properties of living systems and is observed at all levels of biological organization [23–33,109–112], a specific strategy is needed to show the coexistence between different modes of oscillatory behaviour. Much as for multi-stability, demonstrating the coexistence between different oscillatory states can be achieved by showing either the transition to a new periodic state upon suprathreshold perturbation, or the existence of a phenomenon of hysteresis: by changing a control parameter in one direction, and then in the reverse direction, a different oscillatory state should then be observed at the same, intermediate value of the control parameter, in a range bounded by two critical values of this parameter. This would bring evidence that in this range, two limit cycles (birhythmicity) coexist. Such a strategy was implemented to demonstrate birhythmicity in the case of periodically stimulated cardiac cells [36,37] and vocal fold vibrations [46,47]. Much as for multi-stability, the interest of theoretical models is to pinpoint the conditions in which multi-rhythmicity may occur.

One aim of this paper was to provide an overview of molecular mechanisms that underlie the occurrence of multi-rhythmicity. We first briefly reviewed autonomous mechanisms producing birhythmicity or trirhythmicity in models for multiply regulated biochemical systems. To this class belong extensions of a model based on a product-activated allosteric enzyme reaction, which was initially proposed for glycolytic oscillations in yeast and muscle cells (§2.1). We recalled how birhythmicity may arise when this two-variable model is coupled to a process recycling the product into substrate (figure 1*a*). Perturbations in the form of addition of a pulse of substrate of appropriate magnitude allow the reversible switching between the two stable limit cycles, which are separated by an unstable cycle (figure 1*b*), and between the two regimes of sustained oscillations (figure 1*c*). Another extension of the model for glycolytic oscillations consists of the coupling in series of two product-activated enzyme reactions (figure 2*a*). As a result of the interplay between two instability-generating mechanisms, this model is capable of producing simple periodic oscillations, complex periodic oscillations in the form of bursting, chaos, birhythmicity as well as trirhythmicity (figure 2*b*).

Birhythmicity was also found in a model for pulsatile cAMP signalling in *Dictyostelium* amoebae (§2.2) as a result of a competition between two reaction paths within the oscillatory mechanism, and in a model for the *Drosophila* circadian clock (§2.4) when the two branches involved in the negative feedback loop that underlies the oscillations are out of balance. In the case of Ca^++^ oscillations (§2.3) the phenomenon is observed when the mechanism producing a single regime of periodic behaviour is supplemented with a feedback on the input that controls the oscillations. Multiple circuits within a regulatory network are also at the core of the mechanism producing birhythmicity in a model for p53–Mdm2 oscillations [113]. The latter system is of particular physiological significance since the two modes of oscillations might relate to the existence of two oscillatory regimes of p53 and Mdm2 in irradiated cells, characterized by a period of about 6 h or 10 h at low and high doses of irradiation, respectively.

The mechanism for birhythmicity discussed in §2.5 involves the interplay between different oscillatory circuits within a complex regulatory system such as the Cdk network that governs the dynamics of the mammalian cell cycle. The multiplicity of oscillatory circuits within a complex regulatory network can thus provide a source of multi-rhythmicity [66]. Another example of this phenomenon is provided by a simpler model in which the mutual inhibition of two Cdk oscillators governing distinct phases of the cell cycle can give rise to the coexistence between two periodic or chaotic attractors [50].

### 5.2. Coexistence of multiple rhythms or different rhythms in distinct domains of oscillations?

Some oscillatory biological systems can display different rhythmic properties, either in different, closely related conditions or in the same conditions. It is useful to distinguish between these situations, which differ from the point of view of nonlinear dynamics. The distinction can be illustrated by examples taken from the field of neurobiology.

A first example pertains to thalamic neurons. *In vitro* these neurons are capable of exhibiting two different rhythms triggered at different membrane potential levels, which differ by a few millivolts only [114]. The first oscillation occurs, with a frequency close to 10 Hz, at a level slightly depolarized from rest. The second oscillation occurs at a more hyperpolarized level, with a frequency close to 6 Hz. This behaviour can be explained by considering the dynamics of a biochemical system with multiple oscillatory domains [115]. A small change in the value of a control parameter (the polarizing or depolarizing current in the case of thalamic neurons) can cause a switch between the two patterns of oscillatory behaviour. Models pertaining, for example, to reciprocally inhibitory nerve cells [38] show, nevertheless, that different stable patterns of oscillations may sometimes coexist over a large parameter range. A coexistence between different clustering patterns has also been observed in models for small networks of excitatory neurons with heterogeneous coupling strengths [116].

Models for CPGs based on a network of symmetrically coupled oscillatory cells have been proposed to account for the occurrence of different types of gait such as walk, trot, gallop and pace, in quadrupeds [117,118]. The change from one gait to another can be triggered by changes in coupling strengths within a four-cell network [118,119]. The transitions between various gaits appear to originate from the switching between distinct oscillatory domains resulting from a change in some parameter values. Similarly, the switches between different respiratory patterns appear to originate from changes in metabolic or physiological conditions in a model for the CPG controlling breathing [120]. By contrast, a minimal model for a four-cell microcircuit controlling locomotion in *Xenopus* tadpoles [121] predicted regions in parameter space where anti-phase oscillations between left–right centres (swimming behaviour) coexist with in-phase oscillations (synchrony). Such birhythmicity accounts for the long-lasting bouts of synchrony observed experimentally at the start of a swimming episode [121]. The question remains, however, as to the mechanism of the switch between the two oscillatory regimes: is it due to some suprathreshold fluctuation or to a change in the value of a control parameter? A similar coexistence between in-phase and anti-phase oscillations was found in another neurobiological model [40] and in a model for pulsatile insulin release by coupled β-cells undergoing glycolytic oscillations [122].

### 5.3. From multiple entrainment to multi-synchronization

Somewhat similar to the pulsatile stimulation by an exogenous signal is the situation in which a cellular oscillator, e.g. the cell cycle network, is coupled unidirectionally to a second oscillator, such as the circadian clock. Synchronization of the cell cycle and the circadian clock can occur at 1 : 1 or 1 : 2 ratios of frequencies [79,85,86]. Numerical simulations indicate the possible coexistence between different modes of entrainment of the cell cycle by the circadian clock [85].

Experimental evidence indicates that while the cell cycle is coupled to the circadian clock, the latter is also controlled by the cell cycle in various ways. In such a situation, schematized in figure 4*b*, we observed a novel mechanism of ‘multi-synchronization’ for the origin of a new type of multi-rhythmicity. In a previous publication devoted to the bidirectional coupling of the cell cycle and the circadian clock [52], we showed that this coupling favours the robust synchronization of the two oscillators and generally leads to a unique pattern of synchronization. However, in certain conditions, and (so far) only for certain types of coupling, the two oscillators could synchronize in two or even three distinct ways [52]. In §4 we extended these results and built a bifurcation diagram in parameter space to show how birhythmicity or trirhythmicity arise when two or three surfaces corresponding to different modes of synchronization overlap (figure 5). The selection of one or the other modes of synchronization depends on the time at which the bidirectional coupling of the two oscillatory networks begins (figure 9). Although less irregular, the alternation between the evolution to one or another mode of synchronized oscillations is reminiscent of the final state sensitivity reported for multi-rhythmicity in the biochemical model of figure 2*a* [123] and in the case of the unidirectional coupling of the cell cycle to the circadian clock (fig. 8 in [85]). Such sensitivity to initial conditions could be related to the intermingled or rugged attraction basins observed in physical systems admitting multiple attractors [124,125].

The phenomenon of multi-synchronization was first observed by means of numerical simulations in the coupled cell cycle–circadian clock model after we found a discontinuity in the curves showing the synchronization period as a function of a control parameter [52]. Further investigation indicated that the discontinuity was due to the simultaneous presence of two or three periodic solutions corresponding to the coexistence of multiple modes of synchronization. In view of the large number of variables (no less than 62) in the model for the bidirectionally coupled system, we used direct numerical integration to obtain our results, including those that allowed us to build the bifurcation diagrams in figure 5*a*,*b*, One goal for future work will be to search for multi-synchronization in simpler models containing a much smaller number of variables. To this end, we could use simplified versions of models for the circadian clock (e.g. [30]) and the cell cycle [66]. It will be interesting to see whether the reduction in the complexity of these models will nevertheless allow multi-synchronization to occur. Beyond the specific case of the cell cycle and the circadian clock, it would be useful to study the dynamics resulting from the bidirectional coupling of two simpler models for sustained oscillations, so as to determine the minimum requirements for the occurrence of multi-synchronization.

Even if a unique mode of synchronization remains the most common behaviour produced by the bidirectional coupling of the two oscillators, the results on multi-synchronization raise the possibility that multi-rhythmicity may play physiological roles that remain to be uncovered. At the cellular level, one example mentioned above is the phenomenon of mode hopping in NFKB signalling [72] induced by pulsatile TNF stimulation [71], which could control different patterns of gene expression. At the level of organs, an example is provided by the possibility of producing different registers of human voice. Of interest is the observation that interactions between the cardiac and respiratory rhythms may produce spontaneous switches between different patterns of entrainment in the cardiorespiratory system [126]. The question arises as to the unidirectional or bidirectional nature of the interactions between the two physiological rhythms. Another physiological system that could be investigated in regard to the possible occurrence of multi-rhythmicity is the segmentation clock that governs somite formation in embryonic development. This cellular clock involves oscillations in the Notch, FGF and Wnt signalling pathways [127]. A model for the segmentation clock [128] makes it possible to search for the possible occurrence of multi-rhythmicity by varying the strengths of coupling between the three signalling pathways, which can all oscillate on their own. Models are useful in predicting the conditions in which multi-rhythmicity may occur, which is a prerequisite for observing the phenomenon and clarifying its possible physiological roles.

## Data Availability

Data are made accessible through the electronic supplementary material that accompanies this paper. The data are provided in electronic supplementary material [129].
